# Case report: Assessing criminal responsibility and recidivism risk in the behavioral variant of frontotemporal dementia

**DOI:** 10.3389/fpsyt.2024.1437363

**Published:** 2024-07-02

**Authors:** Anouk Karcher, Rayane Hamza, Camille Jantzi

**Affiliations:** ^1^ Centre Universitaire Romand de Médecine Légale (CURML), Hôpitaux Universitaires de Genève (HUG), Geneva, Switzerland; ^2^ University of Geneva, Geneva, Switzerland

**Keywords:** criminal responsibility, forensic psychiatry, frontotemporal dementia, neurodegenerative diseases, neuroprediction, recidivism risk

## Abstract

Frontotemporal dementia (FTD) affects the frontal and temporal lobes of the brain, leading to personality changes, language impairments, and behavioral disturbances, including impulsivity and disinhibition. Assessing responsibility and recidivism risk in forensic evaluations is challenging due to the evolving nature of FTD. Despite limited literature, we present a case of a 45-year-old man with no prior legal or medical history, who committed criminal acts due to behavioral changes linked to the behavioral variant of frontotemporal dementia (bvFTD). Initial assessment found him irresponsible, with a non-evaluable risk of recidivism. Subsequent evaluation showed a low recidivism risk based on clinical evolution. We discuss these findings considering existing literature and Swiss jurisprudence.

## Introduction

Frontotemporal dementia (FTD) encompasses a range of clinical syndromes marked by progressive alterations in behavior, executive function, and language (1). The most frequent clinical phenotype of sporadic and genetic FTD is the behavioral variant (bvFTD) (2). This variant is characterized by changes in personality or behavior, such as behavioral disinhibition, apathy or inertia, loss of sympathy or empathy, perseverative and stereotyped or compulsive and ritualistic behavior, and hyperorality and dietary changes (3). The most pronounced and severe features of bvFTD are disturbances to social and moral decision-making, profound difficulty in following legal and moral rules and norms, and, correspondingly, the commission of social and often legal transgressions (4).

Frontotemporal dementia is a more common cause of early-onset dementia than previously recognized and appears to be more common in men. (5). A study by Johnson et al. from 2005, found that age at onset ranged from 35 to 80 years. Compared with Alzheimer’s disease (AD), the age of onset in FTLD is earlier, with a mean age of onset of about 58 years (6).

While patients with bvFTD may comprehend their actions and occasionally acknowledge their wrongdoing, they exhibit deficiencies in the inhibitory neural circuits of the orbitofrontal, anterior insular, and anterior cingulate cortex, leading to impaired regulation of inappropriate behavior (7). The majority of reported cases of patients with FTD who have committed legal violations concern those still at the onset of their disease, before a definite diagnosis, and still cognitively intact, with a retained knowledge of moral rules and social conventions (8).

## Background

In Switzerland, the assessment of criminal responsibility is guided by specific legal standards and psychiatric evaluations. According to Swiss criminal law, an individual can only be held criminally responsible if they have the capacity to appreciate the unlawful nature of their actions and to act in accordance with that appreciation at the time of the offense. This involves an evaluation of both cognitive and volitional components.

Swiss legislation, particularly the Swiss Criminal Code (SCC), provides the framework for these assessments. Article 19 SCC states that a person is not liable for a criminal act if, at the time of the act, they were incapable of appreciating the wrongfulness of their conduct or of acting according to this appreciation due to a severe mental disorder. If a mental disorder significantly impairs but does not eliminate the individual’s capacity, partial responsibility may be considered, potentially leading to mitigated sentences.

This case report underscores the complex interplay between frontotemporal dementia (FTD) and criminal behavior through the examination of a 46-year-old man, who exhibited criminal acts of extortion and sexual harassment amidst behavioral disturbances associated with the behavioral variant of frontotemporal dementia (bvFTD). Despite the absence of prior medical or legal history, the patient’s abrupt behavioral changes and subsequent criminal actions prompted a comprehensive psychiatric evaluation, leading to the diagnosis of bvFTD. Through a forensic psychiatric examination conducted one year after the offenses and a subsequent re-evaluation two years later, this case report elucidates the challenges in assessing responsibility and recidivism risk in the context of progressive neurodegeneration.

By presenting this case, this study aims to contribute to the understanding of the forensic implications of FTD and to highlight the necessity for a nuanced approach in legal and clinical settings when dealing with individuals exhibiting criminal behavior due to neurodegenerative diseases. The purpose is to provide insights that facilitate informed decision-making regarding responsibility, risk assessment, and appropriate management in similar cases.

## Case

The patient was born in France where he was raised by his two parents. He spent his entire career, over twenty years, working for the same company in Switzerland, without ever encountering any problems. He was also active as president of a sports club and a member of several other associations. He married and had three children with his spouse. The couple never encountered any marital issues until 2019.

The patient has no known medical history, apart from an operation to remove his adenoids when he was four years old. According to the patient medical file and his statement, he has no personal or family psychiatric history. In terms of addictology, he has never used drugs, and his alcohol consumption is moderate (2 to 3 glasses a week). In 2019, changes his behavior were reported by his family. He began to be irritable and even insulting towards his children, for instance by sending them offensive messages. On one occasion, he showed hetero-aggressive behavior by breaking a door. He began exhibiting increasingly impolite behavior, often unaware of the offensiveness of his words. He also began to be more distant, sometimes isolating himself for long periods of time, even during family vacations. Due to his behavior, his wife filed for divorce.

The changes in his behavior also impacted his professional life. He was dismissed one month before the incidents, despite never encountering any difficulties at work over the last three decades. He admitted the existence of a break with the previous state, which he locates in 2019 solely in the professional field. He referred to a lack of motivation at work, for which he was sanctioned by his superior. One month after his redundancy, the patient started blackmailing a former business associate via e-mail, accusing him of illegal activities. He demanded the payment of several thousand Swiss francs under the threat of reporting these activities to the police. Around the same period, he also started making sexual advances by e-mail to a female member of the sports club of which he was president. Penal complaints were filed by the victims shortly after the events. Subsequently, four months after the events, the patient sought psychiatric consultation in response to concerns raised by his family.

At the time of the first psychiatric consultation, he presented a state of psychomotor agitation with logorrhea, flight of incoherent ideas, maladaptive laughter, and considerable emotional detachment. Memory loss was also noted. The psychiatrist suspected a psychotic decompensation with manic features and recommended that he be admitted to a psychiatric ward. The patient was admitted to the hospital four months after the incidents where he remained hospitalized for a total of seven months.

At the time of his admission, the patient’s history quickly led to the suspicion of a neurological disorder. The differential diagnosis of a first psychotic episode in a 46-year-old patient without any particular history must distinguish between a psychiatric disorder (schizophrenia, mood disorder) and a neurological disorder (tumor, cerebrovascular accident, neurodegenerative disorder).

In this context, a cerebral MRI was carried out, which proved reassuring. However, the radiologist’s initial evaluation did not specifically assess for indicators of frontotemporal dementia, and thus this specific differential diagnosis was not considered in the initial interpretation of the imaging.

Neurological investigations were therefore not immediately pursued. After a month-long hospitalization, a neuropsychological assessment revealed cognitive and behavioral changes consistent with the onset of frontotemporal dementia, reflecting patterns observed over the previous year. To further explore the etiology of these symptoms, several exams were carried out. First, a behavioral assessment scale (DAPHNE scale) (9) was performed and scored 5 out of 6 in screening and 12 out of 40 in diagnosis, highlighting the following items: loss of social propriety, inappropriate joviality, loss of initiative, emotional blunting and indifference, attitude with a tendency to persevere, and a change in care and dress.

In light of this information, the brain MRI initially performed was reinterpreted and revealed mild atrophy of the right frontal lobe. The fluorodeoxyglucose (FDG) PET scan showed right frontal hypometabolism, and the DAT-scan revealed left nigrostriatal dopaminergic dysfunction, clinically corresponding to a discrete extrapyramidal syndrome of the right upper limb. The diagnosis of the behavioral variant of frontotemporal dementia was officially established in January 2021, ten months after the incidents.

During his hospitalization, The patient. presented with delusions of persecution with a poisoning theme that motivated the introduction of paliperidone 3mg once daily, which was increased to 6mg due to lack of efficacy. The introduction of antipsychotic treatment led to a reduction in delusions and an improvement in contact. However, as it did not impact the mental disorganization and verbal perseverations, the paliperidone was stopped but had to be restarted at the dose of 3mg as the patient showed more scattered behavior.

Following the diagnosis, outpatient treatment with psychiatric and neurological follow-up was recommended. Weekly ergotherapy sessions were introduced as a particular creativeness was discovered during the occupational therapy sessions in the hospital. According to the neurologist who was treating the patient at the time, a form of artistic rebirth has been described in behavioral variant of frontotemporal dementia. On a therapeutic level, the antipsychotic medication was stopped. Trazodone 50mg was introduced to manage the behavioral symptoms and sleep disorder. Melatonin 6mg was added as an add-on for treating the sleep disorder.

The patient was given a full work incapacity as well as a contraindication to drive a car. A curatorship was set up to support the patient in administrative and financial matters. M.R. moved in with his parents and initially remained capable of maintaining a certain level of autonomy in performing everyday tasks. At the time of the first forensic psychiatric evaluation in March 2021, the patient was living with his parents and was still autonomous for most everyday life tasks. At examination, he presented with motor disinhibition, episodic memory problems, poor and disorganized spontaneous speech marked by frank emotional blunting, and generalized abrasion of affects. His mood was deemed good with no mention of suicidal ideation. The experts highlighted psychotic symptoms, notably delusional utterances with themes of persecution and imaginative and interpretative mechanisms. Hallucinations were not identified by the experts during the interviews. The patient was almost completely anosognosic of his condition.

He was able to declare that, before committing the offenses, he knew that his actions were illegal but decided to favor short-term profits over legal consequences. He even wrote a letter of apology to the first victim and recognized that her complaint was justified. Given that behavioral symptoms were present more than one year prior to the incidents, and that an official diagnosis of FTD had already been established by the time the experts met the defendant, the forensic psychiatrist assessed that the patient. was suffering from frontotemporal dementia at the time of the offenses and that the disorder led him to commit them. Thus, the experts assessed that he was not capable of inhibiting his behavior despite being fully aware of the risks involved mainly because the emotional blunting made him indifferent to this matter. In addition, his judgment was impaired, causing him to focus on the short-term benefits of his actions. Lastly, the loss of social propriety also played a role in the e-mails the patient sent to the female victim. In all, the experts concluded that the offenses were directly caused by the pathology from which the defendant suffered. Consequently, he was found to be irresponsible.

At the time of the first forensic examination one year after the incidents, the experts did not have information on how and how fast the disease would progress. They could only link the risk to the progression of the disease and its consequences and highlight some protective factors such as the neurological and psychiatric follow-up, the supportive and prosocial family environment, and the presence of occupational activities that punctuate his days.

Regarding the evolution of the disease, the experts were commissioned to re-evaluate the patient two years after the incidents and were asked to give a new opinion on the risk of recidivism. By then, the patient was living in assisted care and presented with severe cognitive impairments without behavioral issues. He presented with speech disturbances characterized by verbal stereotypies and was not able to carry out a proper conversation. He needed assistance for most everyday tasks. The experts noted as well the presence of emotional blunting as well as psychomotor retardation. The pharmaceutical treatment then consisted of an antipsychotic treatment, a sedative, as well as a mood stabilizer. It was established that he was unable to use a computer at that time, which was important given the fact that the offenses were committed using a computer. Thus, the overall risk of recurrence has been assessed as low.

## Discussion

The assessment of the recidivism risk was a particular challenge in this case as the neurologists were not able to describe the progression of the disorder. No data was found in the literature regarding these specific pieces of information. The use of standardized risk assessment tools such as actuarial scales or structured clinical judgment was not used in the case of M.R. as there are no recidivism risk assessment scales specifically conceived for the types of offenses he committed (extortion and sexual harassment). The current literature does not provide evidence on how to assess recidivism risk in the case of frontotemporal dementia, or dementia in general. Therefore, the forensic psychiatrists had to rely solely on clinical evidence. Rather than providing an unreliable and uncertain assessment, they chose to only describe the existing risks and protective factors. As the patient did not have a criminal record before committing these offenses and never presented with antisocial behavior, the risk factors were linked to the disease.

Given that the only identifiable risk factor resides in the neurological disorder itself, to reduce the recidivism risk, the experts recommended therapeutic measures adapted to M.R.’s symptomatology. These measures consisted of regular psychiatric and neurological monitoring, occupational activities, curatorship, and a living environment adapted to his level of autonomy.

At the time of the patient’s assessment during his hospitalization, it was crucial for the experts to evaluate the risk of recidivism of sexual behavior towards other vulnerable individuals within the hospital environment. This was deemed more urgent compared to the relatively lower and less dangerous risk of recidivism for extortion. There is an emerging body of literature on the issue of “inappropriate sexual behaviors” (ISB) in the context of major neurocognitive disorders (MNCD) (DSM-5). These behaviors are indeed common and have a significant impact on the individuals’ surrounding environment (10, 11). Although ISB are not yet classified in the DSM-5, Sachdev et al. proposed four criteria in 2017 to define them: the individual must have a diagnosis of MNCD; they must exhibit behavior that is explicitly sexual or perceived as such; the behavior must appear inappropriate; and the behavior must not have preceded the onset of cognitive decline (12).

To ensure a more precise assessment of ISB, a clinical measurement tool was developed by Knight et al.: the Saint Andrew’s Sexual Behaviour Assessment (SASBA) scale. This tool classifies ISB into four categories: verbal comments, no-contact behavior, exposure, and touching others (13). The specific assessment of ISB could have been recommended by the experts for better clinical monitoring of the individual, particularly using the SASBA scale. Such standardized evaluations could provide a foundation for experts to assess the risk of recidivism and dangerousness in the context of dementia.

The initial consideration of a manic episode with organic causes prompts an exploration of related literature. Several studies have demonstrated organic causes underlying psychiatric disorders, as evidenced by the work of Wang and Weiss (14) on frontotemporal dementia, Wallengren et al. (15) on autoimmune encephalitis, Boylu and Kırpınar (16) on myelinolysis, and Soto Ontoso et al. (17) on autoimmune encephalitis. These studies collectively underscore the complexity of diagnosing organic causes of manic episodes and emphasize the significance of interdisciplinary collaboration between neurology and psychiatry in accurate diagnosis and management. Additionally, Lalanne et al. (18) discuss melancholia associated with severe cognitive disorders as an expression of late-onset postpartum anti–N-methyl-D-aspartic acid receptor limbic encephalitis, further highlighting the intricate link between psychiatric symptoms and organic etiologies.

In cases involving organic conditions that present with psychiatric symptoms, the intersection of medical and legal perspectives is particularly critical. Medical experts, including neurologists, neuropsychiatrists, and forensic psychologists, play a crucial role in providing expert testimony regarding the defendant’s diagnosis, prognosis, and the impact of their condition on their mental state and behavior at the time of the offense.

Studies have shown a significant correlation between certain types of dementia and an increase in criminal behavior. Specifically, research by Liljegren et al. (7) found that patients with behavioral variant frontotemporal dementia (bvFTD) were statistically significantly more likely to exhibit criminal behavior and violence compared to patients with Alzheimer’s disease. The most common offenses among the bvFTD group included theft, traffic violations, sexual advances, trespassing, and public urination.

In another study, Diehl-Schmid et al. (19) reported that 54% of patients with frontotemporal lobar degeneration (FTLD), which includes both bvFTD and semantic dementia (SD), exhibited criminal behavior compared to only 12% of patients with Alzheimer’s disease. Most offenses committed by these patients were minor, primarily involving property, with minimal material damage.

According to these studies, individuals with bvFTD exhibit prominent behavioral disinhibition, impulsivity, and social conduct disturbances, which may contribute to their increased likelihood of engaging in criminal acts. In contrast, AD is characterized by progressive memory loss and cognitive decline, with behavioral symptoms typically emerging in later stages. The distinct behavioral profile of bvFTD suggests that its progression may exhibit different characteristics in the context of recidivism compared to AD.

The literature provides several indicators for assessing the disease progression in frontotemporal dementia (FTD). Shorter disease durations are observed in patients with FTD, especially those with genetic mutations such as C9orf72 (20). Key features indicating progression include a positive family history, memory impairment, and clinical abnormalities at presentation (21). Additionally, patients with FTD showing normal MRI results tend to have a more benign course compared to those with atrophy at presentation (22).

The use of neuroimaging for the prediction of recidivism has been described in a few studies (23–25). These studies seek to establish the existence of biomarkers at the cerebral level using structural or functional brain imaging. However, these studies do not focus specifically on neurological disorders. By adding important personalized information about the brain of offenders to the risk assessment equation, we may thereby make it more likely that legal decision-makers rely on the best available tools of violence risk assessment (23). The combination of artificial intelligence (AI) and neuroimaging has led to the development of what can be called” A.I neuroprediction,” which is the use of structural or functional brain parameters coupled with machine learning methods to make clinical or behavioral predictions (26). Potentially, A.I neuroprediction could soon be more generally used to predict the risk of recidivism in forensic psychiatry and criminal justice. However, the application of such techniques raises legal and ethical issues (26).

## Conclusion

This case highlights the difficulties posed by neurodegenerative diseases, and frontotemporal dementia in particular, in forensic psychiatric assessments. These difficulties are all the greater because there is currently no validated or standardized method for assessing the risk of recidivism in these pathologies, and it is not yet possible to determine the evolution of the pathology in each patient. Indeed, we have shown that average life expectancy is reduced in patients diagnosed with the behavioral variant of frontotemporal dementia, but this is only a statistical estimate, and individual variance can be significant.

In recent literature, however, we have seen the emergence of “neuroprediction” techniques, which, by focusing on the identification of neurocognitive markers for the prediction of recidivism, could constitute an avenue for the development of tools for assessing the risk of recidivism. However, these artificial intelligence techniques are at an experimental stage and raise several ethical issues.

The importance of a rigorous assessment of criminal responsibility and the risk of recidivism in patients with frontotemporal dementia is a major issue in psychiatric forensic evaluations. Indeed, these factors are essential in determining their judicial management and pose significant societal challenges, with an error in assessing the risk of recidivism potentially leading to the endangerment of society or the imprisonment of harmless persons. Additional research in this field is warranted.

## Data availability statement

The datasets presented in this article are not readily available because they are contained within a criminal forensic report. Requests to access the datasets should be directed to camille.jantzi@hug.ch.

## Ethics statement

Written informed consent was obtained from the individual(s) for the publication of any potentially identifiable images or data included in this article.

## Author contributions

AK: Writing – review & editing, Writing – original draft. CJ: Data curation, Writing – review & editing, Validation, Supervision, Methodology, Conceptualization. RH: Data curation, Conceptualization, Writing – review & editing.
